# Subretinale Lufteingabe zur Behandlung postoperativer Netzhautfalten nach Ablatio

**DOI:** 10.1007/s00347-021-01485-3

**Published:** 2021-08-30

**Authors:** Viola Radeck, Horst Helbig, Philipp Prahs

**Affiliations:** Augenklinik und Poliklinik, Universitätsklinikum Regensburg, Franz-Josef-Strauß-Allee 11, 93053 Regensburg, Deutschland

**Keywords:** Makulafalte, Netzhautduplikatur, Ablatiochirurgie, Metamorphopsie, Netzhautverlagerung, Macular fold, Retinal fold, Retinal detachment surgery, Metamorphopsia, Retinal shift

## Abstract

**Hintergrund:**

Faltenbildungen der Netzhaut stellen ein Problem v. a. nach Vitrektomie oder Buckelchirurgie mit Gaseingabe dar. Liegen diese Falten im Makulabereich, wirkt sich das meist erheblich auf das Sehvermögen des Patienten aus. Im Folgenden wird eine Behandlungstechnik solcher Falten beschrieben.

**Methode:**

In einem Zeitraum von Januar 2017 bis Juni 2020 wurden 6 Patienten mit der im Folgenden beschriebenen Behandlungstechnik operiert. Es erfolgte die erneute Abhebung der Netzhaut mittels Balanced Salt Solution (BSS), gefolgt von einer subretinalen Eingabe gefilterter Luft. Das Ausstreichen der Falte erfolgte mithilfe von Perfluorcarbon (PFC). Eine postoperative Drainage der subretinalen Luft und Flüssigkeit erfolgte nicht, die Spontanresorption wurde abgewartet.

**Ergebnisse:**

Bei keinem Patienten kam es zu einer Visusverschlechterung nach erneuter Netzhautabhebung. Eine Verbesserung der Sehschärfe zeigte sich bei 4 von 6 Fällen. Eine Verminderung der Metamorphopsien konnte bei 5 von 6 Patienten erreicht werden, 2 Patienten gaben an, die Metamorphopsien gar nicht mehr wahrzunehmen. Nur 1 Patient berichtete über einen gleichbleibend verzerrten Seheindruck trotz anatomisch glatter zentraler Netzhaut.

**Schlussfolgerung:**

Bei kritischer Indikationsstellung stellt unsere Behandlungstechnik eine sichere und erfolgreiche Vorgehensweise zur Behandlung von Netzhautfalten im Bereich der Makula nach Ablatiooperation dar.

## Abstract (en)

**Background:**

Full-thickness retinal folds are an unpleasant complication after vitrectomy or buckle surgery with gas tamponade for the treatment of retinal detachment. If the retinal folds involve the macula, the visual function of the patients will be severely impaired. This article describes a surgical technique for the management of such full-thickness macular folds.

**Method:**

Between January 2017 and June 2020 a total of 6 patients were treated with the following technique. A redetachment was induced with balanced salt solution (BSS), followed by a subretinal air injection with filtered air. The retinal fold was mechanically smoothed out with the aid of perfluorocarbon (PFC). A postoperative drainage of air and BSS was not necessary due to spontaneous resorption.

**Results:**

None of the patients experienced visual loss after redetachment of the retina. The visual acuity improved in 4 of the 6 patients, perception of metamorphopsia improved in 5 out of 6 patients, 2 reported complete disappearance of metamorphopsia and in 1 patient distorted vision was unchanged despite an anatomically smoothed central retina after surgery.

**Conclusion:**

With careful consideration of the indications, this surgical technique has shown to be a safe and promising therapeutic strategy for the treatment of macular full-thickness retinal folds after retinal detachment surgery.

## Hintergrund

Nach einer Operation der Netzhaut bei Ablatio retinae kann es neben einer Verlagerung der Retina zu einer die gesamte Netzhautdicke betreffende Faltenbildung der Netzhaut kommen. Solche Falten treten besonders nach Vitrektomie oder Buckelchirurgie mit intravitrealer Gaseingabe auf [5]. Störend sind diese Falten v. a. dann, wenn sie im Makulabereich liegen. Somit sind v. a. Patienten gefährdet, bei denen die Netzhautablösung bis dicht an die Fovea reicht oder diese gerade überschreitet. Häufig stellen sich Patienten mit Netzhautablösung aber gerade dann beim Augenarzt vor, wenn der wahrgenommene Schatten an oder in das Zentrum reicht.

Eine solche postoperative Netzhautfalte ist besonders ärgerlich, da trotz einer vermeintlich erfolgreichen Operation mit Wiederanlegung der Netzhaut der Patient unter Umständen sehr stark durch Metamorphopsien, Verkleinerungs- oder Vergrößerungseffekte beeinträchtigt ist. Saleh et al. [8] zeigten anhand eines Quality-of-life-Fragebogens, wie stark sich die Patienten durch Metamorphopsien nach Ablatiochirurgie beeinträchtigt fühlen trotz eines guten Visusergebnisses. Auch Fusionsprobleme und Störungen des binokularen Sehens können erhebliche Probleme verursachen.

Verschiedene Maßnahmen wurden zur Prophylaxe der postoperativen Faltenbildung vorgeschlagen. Wesentlich erscheint darunter die vollständige Drainage subretinaler Flüssigkeit und der Verzicht auf eine komplette Gasfüllung. Zur postoperativen Lagerung existieren teils widersprüchliche Empfehlungen: Während manche Operateure die Strategie verfolgen, das Netzhautloch hoch zu lagern, um eine optimale Tamponade des Loches zu erreichen, führt dies in der frühen postoperativen Phase zu einem „Fangen“ der subretinalen Restflüssigkeit am Unterrand der Ablatio, was nach Resorption zur Genese von Netzhautfalten beiträgt. Eine Tieflagerung des Netzhautloches führt zu einem Hängen der Netzhaut auf der Gasblase, was zwar für die Reduktion der Faltenbildung optimal erscheint, möglicherweise aber den Lochverschluss und die Wiederanlegungsrate negativ beeinflussen könnte. Eine Bauchlage tamponiert den hinteren Pol und glättet die zentrale Netzhaut, ist aber für viele Patienten schwer konsequent umsetzbar. Ein sinnvoller Kompromiss erscheint postoperativ eine flache Rückenlage, bei der alle prääquatoriellen Netzhautdefekte tamponiert sind, und gleichzeitig etwas Flüssigkeit auf dem hinteren Pol zu belassen, sodass sich die Makula nach Resorption der verbliebenen zentralen subretinalen Flüssigkeit spontan ohne Druck wieder anlegen kann.

Sind trotzdem postoperative Netzhautfalten entstanden, zeigen sich diese bereits nach Resorption der intraokularen Gasfüllung symptomatisch und erfordern ein schnelles und beherztes Handeln, da ansonsten die Gefahr einer irreversiblen Sehbeeinträchtigung besteht.

Im Folgenden beschreiben wir eine Behandlungstechnik solcher retinaler Falten und zeigen diese anhand von 6 Fallbeispielen aus unserer Klinik.

## Methoden

Zwischen Januar 2017 und Juni 2020 wurden 1968 primäre Ablatiooperationen an der Augenklinik der Universität Regensburg durchgeführt. In dieser Zeit stellten sich 6 Patienten mit Status nach Vitrektomie und Gastamponade bei rhegmatogener Ablatio retinae und symptomatischen Netzhautfalten vor. Alle 6 Patienten klagten über eine erhebliche Beeinträchtigung ihrer Lebensqualität durch Metamorphopsien, und es bestand seitens der Patienten ein dringender Therapiewunsch.

**Figure 1:**
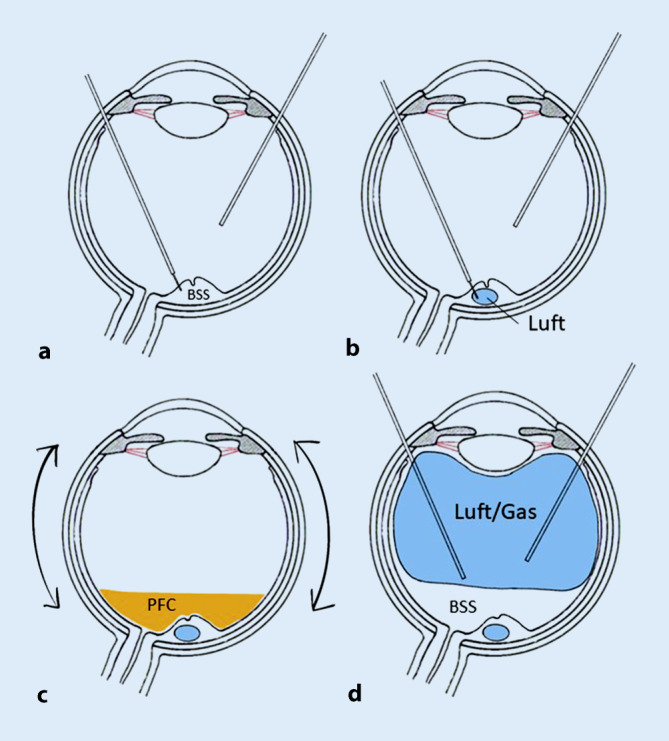

Zur Behandlung der Netzhautfalten nach Ablatiochirurgie wurde folgende Technik angewandt: Im Rahmen einer 23-Gauge-Pars-plana-Vitrektomie wurde zunächst mithilfe einer 41-Gauge-Teflonkanüle im Bereich der Falte subretinal BSS eingegeben, um die Netzhaut im Bereich des hinteren Pols erneut abzuheben (s. Abb. 1a). Die Einstichstelle wurde dabei primär entfernt vom Zentrum im Bereich der Gefäßbögen ausgewählt.

Bei Bedarf wurde an einer 2. und 3. Stelle entlang der Falte subretinal BSS eingegeben, bis in einer konfluierenden Blase die subretinale Flüssigkeit den gesamten hinteren Pol einschließlich der Netzhautfalte erneut abgehoben hatte. Die komplette Abhebung des hinteren Pols war nicht immer primär erfolgreich, da sich die subretinal injizierte Flüssigkeit nicht immer konzentrisch um die Injektionsstelle ausbreitete, sondern longitudinal entlang der Falte nach peripher floss und von der Adhäsionskraft zwischen Fotorezeptor und Pigmentepithel abhing. In einem derartigen Fall wurde versucht, durch eine vorübergehende Lufteingabe in den Glaskörperraum die subretinalen Flüssigkeitsblasen zum Konfluieren in der Makula zu bringen.

Bei allen 6 Patienten war die einfache Abhebung mithilfe von subretinaler Injektion von Balanced Salt Solution (BSS) nicht ausreichend, um die Falten zu glätten. Es erfolgte bei allen 6 Patienten anschließend eine Eingabe von gefilterter Luft (bis zu 100 µl) in die subretinale BSS-Blase (s. Abb. 1b), hierbei wurde dieselbe Mikroretinotomie verwendet. Um einen Gegendruck zur Glättung der Netzhaut von innen zu erreichen, wurde epiretinal Perfluorcarbon (PFC) auf den hinteren Pol aufgegeben (s. Abb. 1c). Durch die Oberflächenspannung der subretinalen Luftblase im Bereich der äußeren Netzhautschichten und gleichzeitigem Gegendruck durch die Decalin-Blase auf die innere Netzhautschicht gelang es bei allen Patienten, durch vorsichtige Bulbusbewegungen die Netzhautfalten auszustreichen. Luft und BSS wurden subretinal belassen.

Im Anschluss erfolgte der Austausch von Decalin und BSS im GK-Raum gegen Luft (s. Abb. 1d). Hierbei wurde darauf geachtet, dass der hintere Pol nicht komplett trockengelegt wurde, sondern dass ca. ein Viertel bis die Hälfte des Glaskörpervolumens mit BSS verblieb. Dies ermöglicht der Netzhaut, durch Resorption der subretinalen Flüssigkeit über das RPE eine faltenfreie Anlage zu erreichen. Dann erfolgte der Austausch von Luft gegen Gas (20 % SF6). Bei einem Patienten (Fallbeispiel 5) entschied man sich abschließend für eine intraokulare Luftfüllung und verzichtete auf den Austausch gegen Gas (SF6). Postoperativ wurde bei allen Patienten Rückenlage mit flachem Kopf verordnet.

Die vorliegende Studie wurde durch die lokale Ethikkommission genehmigt.

## Ergebnisse

Die oben beschriebene Operationstechnik wurde bei 6 Patienten mit zentralen Falten nach Ablatiooperation angewandt. Die wesentlichen Daten sind in Tab. 1 dargestellt.

**Table 1:** 

Fallbeispiel	Alter/Geschlecht	Ablatioverlauf/Grenze	Visus vor/nach Revision	Metamorphopsien vor/nach Revision	Zeitpunkt Revision (Wochen nach primärer Ablatiooperation)
1	54/m	Hochbullös von temporal oben/Fovea gerade angespült	0,8/0,8	Ja/nein	4
2	47/m	Flache Ablatio von temporal oben/Fovea gerade angespült	0,6/0,6	Ja/ja (weniger)	6
3	50/m	Hochbullös von oben/Fovea gerade angespült	0,4/0,5	Ja/ja (gleich)	5
4	68/w	Hochbullös von oben/Fovea gerade angespült	0,4/0,6	Ja/nein	4
5	63/m	Hochbullös von oben/Fovea gerade angespült	0,4/0,8	Ja/ja (deutlich weniger)	8
6	65/w	Hochbullös von oben/Fovea gerade angespült	0,5/0,8	Ja/ja (deutlich weniger)	5

Bei allen Patienten war bei frischer, mobiler Ablatio von temporal oder oben mit einer zentralen Ablatiogrenze gerade in der angespülten Fovea eine primäre 23-Gauge-Vitrektomie mit PFC, Laser oder Kryo des Risses und einer 20 % SF6- oder 16 % C2F6-Gastamponade durchgeführt worden. Bei allen Patienten bis auf Patient Nr. 6 lautete die Lagerungsempfehlung für die ersten Tage nach der Ablatiooperation Seitenlage auf der nicht operierten Seite oder erhöhte Kopflage, um den ursächlichen Riss temporal oder oben durch die Gasblase zu tamponieren. Patient 6 sollte postoperativ flach auf dem Rücken liegen.

Nach Resorption der Gasblase zeigte sich bei allen 6 Patienten eine anliegende Netzhaut, aber auch das Vorhandensein eine Netzhautfalte in der Fovea. Die Patienten beklagten erhebliche subjektive Beeinträchtigung durch Verzerrtsehen. Nach ausführlicher Aufklärung über die Optionen, entschieden sich die Patienten für eine Revisionsoperation.

**Figure 2:**
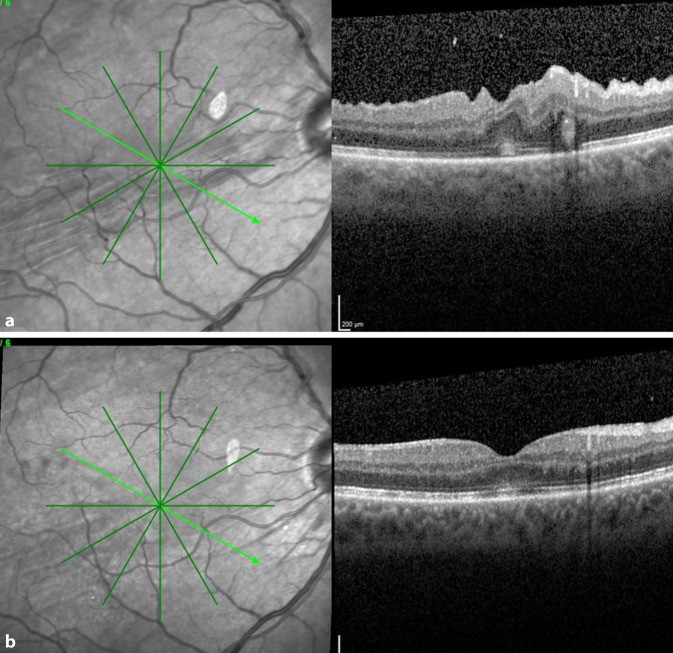

Nach der Revisionsoperation zeigte das postoperative OCT bei allen Patienten eine deutliche Besserung oder vollständige Glättung der ehemaligen Falten. Die Abb. 2, 3 und 4 zeigen die OCT-Bilder vor und nach Revisionsoperation bei 3 Patienten. Bei 4 von 6 Patienten zeigte sich ein besserer Visus nach der Operation der Netzhautfalte, bei den beiden anderen blieb der Visus mit 0,6 bzw. 0,8 auf hohem Niveau stabil. Zwei Patienten hatten postoperativ keine störenden Metamorphopsien mehr. Bei 2 weiteren besserte sich das Verzerrtsehen. Bei 1 Patienten waren die Metamorphopsien trotz wesentlich gebessertem OCT-Befund subjektiv unverändert störend.

**Figure 3:**
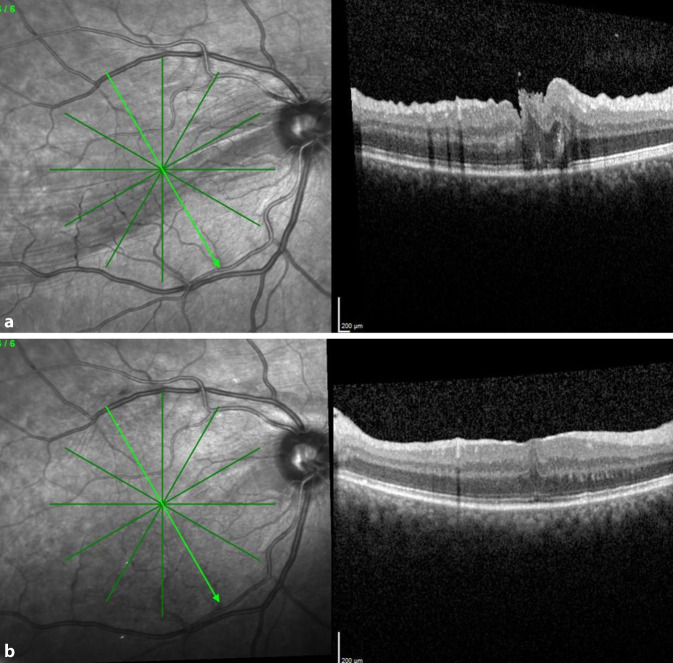

**Figure 4:**
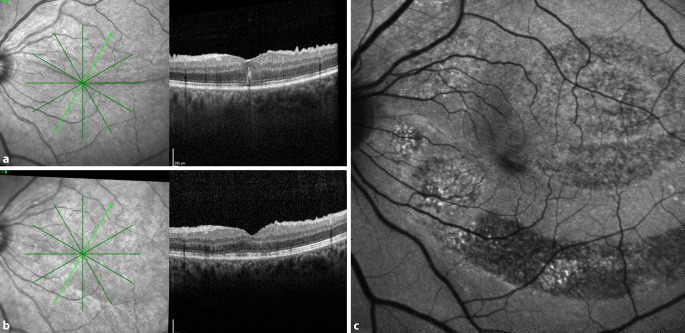

Die Abb. 4 zeigt eine Autofluoreszenzaufnahme nach der Revisionsoperation. Deutliche Störungen der Autofluoreszenz sind in den Bereichen zu erkennen, in denen die Abhebung der Makula mit Injektion von subretinalem BSS erfolgte. Dennoch besserten sich Visus und Metamorphopsien bei dem Patienten. Die Autofluoreszenzbilder der restlichen Patienten zeigten nur geringe Unregelmäßigkeiten im Bereich der Injektionsstelle.

## Diskussion

Eine Makulafalte über die gesamte Netzhautdicke („full-thickness macula fold“) nach Operation einer Netzhautablösung ist neben den häufiger auftretenden Netzhautverlagerungen ohne Faltenbildung ein zwar selteneres, jedoch sehr beeinträchtigendes Ereignis, welches die Freude über die vermeintlich gelungene Wiederanlegung der Netzhaut trübt. In unserer Fallserie war auffällig, dass es sich bei allen Netzhautablösungen um eine mobile Form von oben oder temporal oben mit Makulabeteiligung handelte, die Ablatiogrenze verlief dabei gerade bis über die Fovea. Weiterhin zeigte sich, dass bei 5 von 6 Fällen die Lagerung „Oberkörper hoch“ oder Lagerung auf der nicht operierten Seite empfohlen wurde. Einer Patientin wurde „flache Rückenlage“ empfohlen. Während Schawkat et al. [9] in einer randomisierten Studie keinen Effekt der postoperativen Bauch- oder Rückenlage auf die Makulaverlagerung und postoperative Metamorphopsien fanden, beschrieben Casswell [2] weniger Netzhautverlagerungen nach postoperativer Bauchlage im Vergleich zur Lagerung zur Tamponade des Risses, aber keinen Unterschied in Visus oder Wahrnehmung von Verzerrungen. Das optimale Vorgehen zur Faltenvermeidung durch postoperative Lagerung erscheint somit noch unklar.

**Figure 5:**
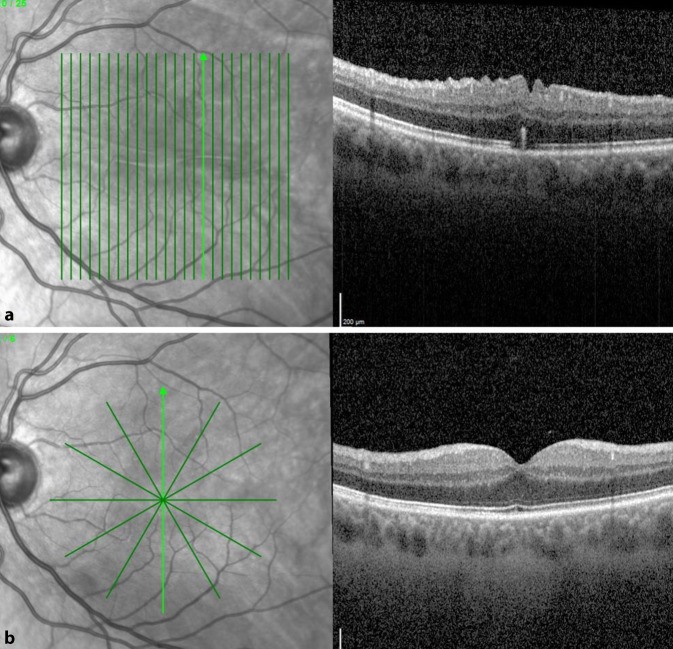

Die Entscheidung zur operativen Intervention bei postoperativen Netzhautfalten ist nicht einfach, und es gibt keine eindeutigen Empfehlungen der Fachgesellschaften [5]. Spontane Besserungen sind beschrieben, und auch wir haben Fälle beobachtet, bei denen sich die Falten und die Metamorphopsien ohne Intervention gebessert haben. Die Abb. 5 zeigt ein solches Beispiel, bei welchem eine anfangs deutlich erkennbare Falte nach 2 Jahren vollständig verschwunden war. Die spontane Prognose scheint bei Falten, die nur die Außen- oder Innenschicht der Netzhaut betreffen, besser zu sein. Bei Falten, die die gesamte Netzhautdicke betreffen, und die zu einem Kontakt von Fotorezeptoren in der Falte führen, scheint die Chance auf eine spontane Besserung geringer.

Im Tierversuch zeigten sich schon nach wenigen Wochen bei experimenteller Faltenbildung der Netzhaut histologisch erhebliche Degenerationen der äußeren Netzhaut [4]. Daher sollte die Entscheidung zu einer operativen Revision rasch erfolgen.

Verschiedene chirurgische Techniken wurden für die Glättung postoperativer Netzhautfalten nach Ablatio beschrieben. Gemeinsam ist den meisten Methoden die Abhebung der Netzhaut im Bereich der Falte. Herbert et al. [6] beschrieben eine subretinale Injektion von BSS mit einer 41-Gauge-Kanüle und temporäre Eingabe einer schweren Flüssigkeit (F6H8). Postoperativ wurde Rückenlage verordnet und das F6H8 nach 5 Tagen wieder entfernt.

El-Amir et al. [3] beschrieben ebenfalls eine Abhebung mittels subretinaler Injektion von BSS im Bereich der Falte, aber anschließend intravitreale Gaseingabe und postoperative Gesichtstieflage. Diese Methode wurde auch von Heimann und Bopp favorisiert. Trinh et al. [10] modifizierten die Methode durch eine Silikontamponade des Glaskörperraums.

In einem Case Report einer französischen Arbeitsgruppe wurde die Technik einer erneuten Netzhautabhebung und anschließender Drainage über ein iatrogenes Foramen temporal inferior beschrieben [7]. Dies erscheint jedoch nicht obligat und birgt möglicherweise ein erhöhtes Risiko für eine PVR-Reaktion.

Die komplette Abhebung der Makula durch subretinale Injektion von BSS mittels 41-Gauge-Teflonkanüle gelingt in der Praxis nicht immer mühelos. Witkin und Hsu [11] erreichten ein Konfluieren mehrerer Blasen subretinaler Flüssigkeit und ein Öffnen der Falte erst nach 5 min unter Luft im Glaskörperraum. El-Amir beschrieb eine longitudinale Ausbreitung der subretinalen Flüssigkeit entlang der Falte und keine konzentrische subretinale Blase um die Einstichstelle [3]. Bei starren Netzhautfalten ist die subretinale BSS-Injektion nicht ausreichend. El-Amir beobachtete nach unabsichtlicher Luftinjektion eine Öffnung einer starren Falte [3]. Ausgehend von ihrer Erfahrung mit der subretinalen Injektion im Rahmen einer Gentherapie der Netzhaut beschrieben Barale et al. die geplante subretinale Injektion von Luft bei Netzhaufalten [1].

Die subretinale Eingabe von Luft stellt nach unseren Beobachtungen den effektivsten Schritt zur Entfernung der Duplikatur der äußeren Netzhautschichten dar. Beim Versuch, die Netzhautfalte allein durch Eingabe von BSS subretinal zu entfernen, zeigt sich regelhaft ein quaddelförmiges Ausbreiten des Flüssigkeitsvolumens nach lateral, da die Adhäsionskräfte von Netzhaut und Pigmentepithel weniger stark ausgeprägt zu sein scheinen als der Zusammenhalt zwischen den verklebten äußeren Netzhautschichten im Bereich der Falte. Durch Eingabe von zusätzlichem Volumen erhöht sich der subretinale Druck, und es trennen sich erneut lediglich Netzhaut und Pigmentepithel im Randbereich der Ablösung. Da es sich ausnahmslos um Augen handelte, die erst kürzlich eine rhegmatogene Netzhautablösung hatten, ist zu erwarten, dass der mechanische Zusammenhalt zwischen Netzhaut und Pigmentepithel noch nicht vollständig wiederhergestellt ist, was den beschriebenen Effekt zusätzlich verstärkt.

Mit der Eingabe von subretinaler Luft entsteht eine Phasengrenze im subretinalen Raum. Dadurch wirkt zusätzlich zum subretinalen, hydrostatischen Druck die Grenzflächenspannung zwischen Luft und dem zum großen Teil aus Wasser bestehenden Netzhautgewebe. Diese Grenzflächenspannung weist einen idealen, tangential zur Netzhaut verlaufenden Kraftvektor auf und verhindert regelhaft eine laterale, quaddelförmige Ausbreitung der Luftblase. Durch den Effekt der Grenzflächenspannung tendiert die Luftblase – ähnlich wie beim Aufblasen eines Luftballons – zum Annehmen der Kugelform, was für die Elimination der Falte von Vorteil ist.

Die oben genannten Überlegungen sind zutreffend, solange die Luftblase im Bereich unter der Netzhautfalte gehalten werden kann. Bei sehr geringer Netzhautadhäsion kann diese bei Bulbusbewegungen bis zur Ora aufsteigen. Eine Lösung besteht hier im partiellen Austausch von Flüssigkeit gegen Luft im Glaskörperraum, was das subretinale Aufsteigen von Luft nach peripher und anterior verhindert, allerdings wird die optische Qualität des Einblicks durch Reflexionen und Verzerrungen an den Grenzflächen in der Folge reduziert.

Ein begrenzter Gegendruck zum Manövrieren der subretinalen Luftblase kann auch durch epiretinales Perfluorcarbon (PFC) erfolgen. Die akzidentelle Eingabe von multiplen kleinen Luftblasen in den Subretinalraum kann bei der hier beschriebenen Zielsetzung sogar von Vorteil sein, da die Grenzflächenspannung umgekehrt proportional zum Radius der Blase ist. So maximiert eine kleine Luftblase, solange sie unter der Falte positioniert werden kann, die tangentiale, faltenentfernende Kraft auf die Netzhaut.

Durch eine Massage der Netzhaut von 2 Seiten, von innen mit Luft und von außen mit PFC, sowie durch gezielte intraoperative Rotation des Bulbus, lassen sich auch hartnäckige steife Falten öffnen und die Makula glätten.

Die Operationstechnik von Barale et al. haben wir in der vorliegenden Arbeit modifiziert. Barale et al. propagieren, nach der subretinalen Eingabe von Luft und Balanced Salt Solution (BSS) und abschließender Netzhautglättung das subretinale BSS und die Luft wieder zu entfernen und dazu ein Drainageloch anzulegen [1]. Dies ist unserer Erfahrung nach nicht notwendig. Die subretinale Luftblase zeigte sich in allen Fällen spätestens am 2. postoperativen Tag vollständig resorbiert. Ein Anhalt für eine langfristige funktionelle Schädigung der Fotorezeptoren durch ein Belassen der submakulären Luft ergab sich in den hier beschriebenen Fällen nicht. Der Visus blieb stabil oder besserte sich. Dem gegenüber steht die Tatsache, dass bei einer sofortigen Wiederanlegung und intraoperativen Drainage der Luft ein zusätzlicher recht zentraler Netzhautdefekt zur Drainage benötigt wird, der wiederum mit einer möglichen Induktion einer PVR-Reaktion oder einer Bildung epiretinaler Membranen einhergehen kann.

Dass diese Manöver zur Abhebung der Netzhaut nicht völlig atraumatisch sind, zeigte die postoperative Autofluoreszenz (Abb. 4). Zwar konnten diese ausgeprägten Veränderungen in der Autofluoreszenz bei den restlichen Patienten nicht in diesem Ausmaß nachgewiesen werden, aber auch hier sah man postoperativ dezente Unregelmäßigkeiten im Bereich der Injektionsstelle. Auch Zacharias beschrieb nach Abhebung der Netzhaut mit BSS deutliche Veränderungen in der Autofluoreszenz, vermutlich durch ein mechanisches Trauma der forcierten subretinalen Flüssigkeitsinjektion [12]. Dennoch hatten alle Patienten in der vorliegenden Studie eine positive Visusentwicklung.

## Schlussfolgerung

Wir empfehlen das oben genannte Vorgehen als neue intraoperative Option bei starren Makulafalten, die direkt die Fovea mit einbeziehen und sich nach Eingabe von subretinalem BSS nicht glätten lassen. Der Patient sollte vorher gründlich über die Risiken einer erneuten Abhebung der Makula aufgeklärt sein und die Indikation für einen solchen Eingriff sehr kritisch gestellt werden, abhängig vom Befund, aber auch von den subjektiven Beschwerden des Patienten. Weiterhin sollte gründlich darüber aufgeklärt werden, dass postoperativ eine Persistenz der Metamorphopsien bestehen kann.

Eine postoperative Netzhautfalte kann erst nach Resorption des intraokularen Gases nach der primären Ablatiooperation beurteilt werden. Sollte sich dann nach Abwägen aller Risiken eine Indikation zur Revisionsoperation der Falte ergeben, sollte diese möglichst zügig durchgeführt werden, da ansonsten das Risiko einer bleibenden Sehbeeinträchtigung sowie einer Zunahme der Rigidität der Falte besteht.
